# Virtual reality applications based on instrumental activities of daily living (iADLs) for cognitive intervention in older adults: a systematic review

**DOI:** 10.1186/s12984-023-01292-8

**Published:** 2023-12-19

**Authors:** Jorge Buele, José Luis Varela-Aldás, Guillermo Palacios-Navarro

**Affiliations:** 1 SISAu Research Group, Facultad de Ingeniería, Industria y Producción, Universidad Indoamérica, Ambato, Ecuador; 2 Centro de Investigaciones de Ciencias Humanas y de la Educación (CICHE), Universidad Indoamérica, Ambato, Ecuador; 3 Department of Electronic Engineering and Communications, University of Zaragoza, Teruel, Spain

**Keywords:** iADL, Mild cognitive impairment, Cognitive rehabilitation, Cognitive functions, Older adults, Virtual reality

## Abstract

**Background:**

In recent years, the use of virtual reality (VR) as a complementary intervention in treating cognitive impairment has significantly increased. VR applications based on instrumental activities of daily living (iADL-VR) could offer a promising approach with greater ecological validity for intervention in groups with cognitive impairments. However, the effectiveness of this approach is still debated.

**Objective:**

This systematic review aims to synthesize the effects of iADL-VR interventions to rehabilitate, train, or stimulate cognitive functions in healthy adults and people with mild cognitive impairment (MCI) and different types of dementia.

**Methods:**

A systematic search was performed in the Scopus, PubMed, IEEE Xplore, Web of Science, and APA PsycNet databases until September 2022 and repeated in April 2023. The selected studies met the search terms, were peer-reviewed, included an iADL-VR intervention, and were written in English. Descriptive, qualitative studies, reviews, cognitive assessment, non-intervention studies, those unrelated to VR or iADL, those focused on motor aspects, and non-degenerative disorders were excluded. The PEDro scale was used to assess the methodological quality of the controlled studies. To present and synthesize the results, we organized the extracted data into three tables, including PEDro scores, participant characteristics, and study characteristics.

**Results:**

Nineteen studies that met the inclusion and exclusion criteria were included. The total sample reached 590 participants, mostly women (72.67%). Approximately 30% were diagnosed with Alzheimer’s disease or dementia, and 20% had mild cognitive impairment. Variables such as authors and year of publication, study design, type of intervention and VR applied, duration of the intervention, main findings, and conclusions were extracted. Regarding demographic characteristics, the sample size, age, sex, years of education, neurological diagnosis, dropouts, and the city and country where the intervention took place were recorded. Almost all studies showed improvements in some or all the outcomes after the intervention, generally greater in the iADL-VR group than in the control group.

**Conclusion:**

iADL-VR interventions could be beneficial in improving the performance of cognitive functions in older adults and people with MCI and different types of dementia. The ecological component of these tasks makes them very suitable for transferring what has been learned to the real world. However, such transfer needs to be confirmed by further studies with larger and more homogeneous samples and longer follow-up periods. This review had no primary funding source and was registered with PROSPERO under registration ID: 375166.

**Supplementary Information:**

The online version contains supplementary material available at 10.1186/s12984-023-01292-8.

## Introduction

Cognition encompasses various intellectual functions and processes, including perception, attention, consciousness, language, memory, emotions, and executive functions [1, 2]. These cognitive functions work in conjunction with visuospatial abilities, which make it possible to identify stimuli necessary for movement, depth perception, and environmental navigation [3]. These cognitive abilities are essential for individuals to carry out their routine tasks and maintain normal functioning in their environment. Everyday life tasks and routines are called activities of daily living (ADLs) and are essential for self-care and independence [4]. They can be classified into two subgroups: basic activities of daily living (bADL) and instrumental activities (iADL) [5]. Personal care activities such as bathing, dressing, toileting, and functional mobility (the ability to move from one place to another) are in the bADL group [6]. Instrumental activities are based on more complex skills that require multiple cognitive processes, such as food preparation, medication management, and financial management.

According to the American Occupational Therapy Association (AOTA), there are 11 iADLs that are more complex and, as such, may present a challenge for some older adults [7]. These include tasks such as (i) care of others; (ii) care of pets and animals; (iii) child rearing; (iv) communication management; (v) driving and community mobility; (vi) financial management; (vii) home establishment and management; (viii) meal preparation and cleanup; (ix) religious and spiritual expression; (x) safety and emergency maintenance; and (xi) shopping [7]. A decrease in the ability to perform iADLs is linked to the initiation of cognitive decline, which can be a component of the natural aging process. The decline in iADL performance becomes increasingly noticeable as MCI emerges and becomes even more pronounced in cases of dementia [8–10]. As the world’s population ages, the incidence of dementia increases, a term that encompasses a variety of clinical diagnoses, including Alzheimer’s disease (AD), Parkinson’s disease, vascular dementia (VaD), frontotemporal dementia (FTD), Huntington’s disease and Lewy Body Dementia (LBD), each with their own causes and specific risk factors. As pointed out by Iribarnea et al., [11] AD is the most common cause of dementia, accounting for 80% of cases in people over 65 years of age, followed by VaD, FTD and LBD. Zhang et al. [12] pointed out in their review that Mild AD is the leading cause of dementia, accounting for 50–70% of cases. In another study, Iadecola et al. [13] highlighted that although AD prevails as the leading cause of clinically diagnosed dementia in Western countries, VaD might be more prevalent in East Asia. Furthermore, several vascular factors emerge as important factors in the pathogenesis and clinical manifestation of AD [14].

Alzheimer’s disease is believed to be linked to an abnormal buildup of proteins in the brain, such as beta-amyloid and tau protein, which leads to degeneration of brain cells [15]. This disease progresses in stages, beginning with the impairment of episodic memory, followed by deficits in areas such as semantics and attention, and later, with deficits in visuospatial and auditory-verbal memory [16]. The increase in the prevalence of neurodegenerative diseases, such as Alzheimer’s disease, has become a global phenomenon. In 2019, more than 50 million people worldwide were estimated to be living with dementia, and this number is expected to increase to 150 million by 2050, posing a significant burden on healthcare systems and society in general [17].

Given the increasing prevalence of neurocognitive disorders, interventions to improve cognitive functions and iADL are of great importance [18]. These interventions include cognitive training (CTR), which focuses on the systematic practice of specific tasks to improve cognitive performance [19]; cognitive rehabilitation (CRE), which addresses specific cognitive difficulties through compensatory and adaptive strategies [20]; and cognitive stimulation (CST), which involves participation in a variety of cognitive and social activities to maintain and improve overall cognitive functioning [21]. Paper-and-pencil therapies represent traditional cognitive treatments that have been used for several decades to address cognitive deficits in older adults. Although they are beneficial and clinically validated, they present difficulties in maintaining patient motivation and adherence to these processes [22]. In addition, the lack of ecological proposals in clinical neuropsychology interventions has been criticized because they do not always reflect the actual functional performance of the individual [23]. Ecological validity is used in this context to express generalizability (the level to which the findings of an evaluation relate to and/or predict behaviors beyond the testing environment) and representativeness (the plausibility or degree to which the evaluations resemble situations of daily life in which such behaviors will be necessary) [24].

VR has emerged as a potentially valuable tool in the field of cognitive intervention, offering real and ecologically valid demands to stimulate neuroplasticity and enhance regenerative processes, as established by Maggio et al. [25]. In this sense, neuroplasticity refers to the brain’s ability to change and adapt in response to environmental experiences and stimuli [26]. Stimulation of these areas can strengthen neural connections and improve information processing capacity, which can lead to improvements in cognitive performance and the ability to perform iADLs. Therefore, understanding neuroplasticity is essential to explain how behavioral interventions and VR can improve cognitive functions in older adults with cognitive impairment. Shah et al. [27] mention that cognitive training can stimulate neuroplasticity, thus increasing cognitive reserve.

The existing literature suggests that VR could be beneficial in the evaluation and intervention of dementia and MCI [28]. Skurla et al. [29] investigated the relationship between VR and mental health in older adults and mentioned that it can be used as a training tool, although there are still areas for potential improvement. Coyle et al. [30] presented a systematic review showing that computerized and VR training had consistent improvements in attention, executive function, memory (visual and verbal), and memory strategy. There were also favorable psychological effects, including a reduction in depressive symptoms and anxiety. Papaioannou et al. [31] reviewed the efficacy and moderators of VR for cognitive training in people with dementia and mild cognitive impairment, suggesting that VR is an effective treatment in this population. Maggio et al. [28] presented a scoping review that highlighted the opportunities and challenges in the implementation of VR technology. They emphasized the possibility of increasing motivation and participation, which could improve the effects of conventional therapies. Likewise, Yu et al. [32] investigated the use of virtual and augmented reality technologies in neuropsychological rehabilitation and underlined the potential of these technologies to improve quality of life and cognitive performance in older adults.

It is important to mention that VR can come in different degrees of immersion, such as non-immersive, semi-immersive, and fully immersive. At the non-immersive level, virtual environments are presented from a conventional computer, and users control their interaction through devices such as joysticks or other controllers [33]. There is an intermediate category of VR known as semi-immersive VR, where users interact with the virtual environment, but are still aware of their surrounding physical environment. Although perception of the real world is not completely blocked, semi-immersive systems can offer an immersive experience with interactive features [34]. This type of VR can come with more sophisticated graphics and larger flat screens or a large screen projector [35], although we can also find the use of the IREX system, which combines a monitor, video camera, virtual objects, and data gloves to recognize the movement of patients [36]. On the other hand, fully immersive VR allows users to experience a simulated reality in an immersive way, giving them the feeling of living inside the virtual world [37]. Fully immersive VR systems can include projections on surrounding physical surfaces or even head-mounted displays (HMDs) that completely immerse the user in the virtual environment, achieving a high degree of immersion [38].

However, excessive use of HMD can lead to unwanted effects, including visual disturbances, disorientation, postural instability, nausea, headache, and postural discomfort, among others [39]. These effects are explained by conflicts in sensory and spatial integration. In the way virtual environments are designed, there is often a mismatch between the visual system, the vestibular system, and the individual’s movement or posture system. The user receives visual signals of movement, while their vestibular system indicates that there is no change in posture or actual movement. When the individual cannot quickly integrate this information, which differs from his or her experience in the real world (even if it is simulated), discomfort and physiological problems may arise. Furthermore, the lack of synchronization between virtual images, motion detection through the helmet and integration with corresponding visual feedback can lead to orientation problems and dizziness [40]. Importantly, when users have health problems, such as a history of epileptic seizures, the risk of adverse effects increases significantly. Therefore, it is essential to evaluate the health history of participants before including them in an intervention that involves the use of VR.

The review carried out by Corregidor-Sánchez et al. [41] analyzed the effectiveness of virtual systems (not specifically ADL-based interventions) in improving the performance of older people in carrying out their daily activities. They analyzed 23 studies and found a slight improvement effect on iADL, although not significant. It is also mentioned that the quality of the evidence from these studies is very low, and therefore, their true contribution is uncertain, motivating the development of studies with higher quality and methodological rigor. Kurz did something similar, dividing VR applications into three categories: stimulation, training, and cognitive rehabilitation [42]. He concluded that further randomized controlled trials (RCTs) are needed to validate delayed cognitive decline and its positive influence on ADLs and quality of life. On the other hand, in the review carried out by Romero-Ayuso et al. [43], VR applications based on iADL were used in cognitive screening and assessment. The authors highlighted several key advantages of VR, including its cost-effectiveness, safety features, ability to authentically replicate real-world scenarios (ecological validity), versatility in addressing diverse conditions, and the convenience it offers in terms of seamless data collection and scoring [44].

Although there are challenges and limitations associated with VR, its use in interventions could significantly improve the quality of life for affected individuals and their caregivers. The literature search did not identify any previous reviews that specifically focused on the analysis of VR applications that simulate activities of daily living in interventions for older adults with degenerative cognitive disorders. Given the importance of iADL for older adults and the need to find effective interventions to maintain and improve their cognitive functions, it is relevant to address this research topic. The purpose of this systematic review is to synthesize existing evidence regarding the effectiveness of cognitive intervention strategies that use VR to simulate iADL for intervention in healthy older adults, those with MCI, or those with dementia. We hypothesize that continuous training with iADL-based VR applications will have a satisfactory effect on improving comprehensive cognitive function. As secondary objectives and considering the knowledge acquired in this review, we will seek to provide guidelines or instructions for clinical practice and the implications that this review may have in future research, indicating where future research on iADL based on VR should be directed.

## Methods

### Search strategy

One of the authors (JB) conducted a systematic computerized search in the electronic databases: Scopus, PubMed, IEEE Xplore, Web of Science, and APA PsycNet. The keywords and search syntax were adapted to the characteristics of each database. The exact search terms are included in Additional file 1: Annex 1. A comprehensive search of the English-language literature was performed, and the range of years was considered from inception to April 20, 2023. This review was registered in PROSPERO (Registration ID: 375166).

### Inclusion criteria

Studies were included if they a) were original peer-reviewed articles, including controlled studies or single-group studies; b) involved an iADL-VR intervention for training, rehabilitation, and/or stimulation of cognitive functions; c) had at least one outcome measure related to the clinical effects obtained after the intervention; and d) were published in English.

Regarding VR systems, all types were admitted; however, immersive, semi-immersive, and non-immersive systems were differentiated. According to the literature, applications based on immersive VR systems could have greater ecological validity [45, 46]. There were no restrictions on the age, sex, years of formal education, dropouts, or nationality of the selected study participants. There were no limitations regarding the program’s administration, frequency, duration, intensity or sessions. For interventions that included an experimental group (EG) using VR and a control group (CG), both active (ACG) (other interventions) and passive (PCG) (no procedure) controls were included.

### Exclusion criteria

We established the following exclusion criteria for study selection: a) studies solely focused on theoretical, descriptive, or qualitative research without providing any type of intervention; b) studies focused solely on motor rehabilitation or if they aimed at cognitive-motor rehabilitation; c) studies that considered cognitive disorders that are not degenerative in nature, such as stroke or traumatic brain injury (TBI); d) studies involving cognitive screening or diagnosis; e) interventions not aimed at cognitive training and rehabilitation; f) studies not involving the use of VR or not including ADLs in the intervention; and g) any type of review, including both systematic and narrative reviews, as well as meta-analyses.

### Data extraction

Data from the included articles were extracted by two reviewers (JB and JV-A) who worked independently. The general characteristics and results of the studies were recorded, including the author’s name, the year of publication, the study design, the type of intervention and VR applied, the task or place simulated virtually, the duration of the intervention, main results, and conclusions. Regarding the demographic characteristics of the participants in the different studies, the following variables were recorded: sample size, mean age and standard deviation, sex, years of formal education, neurological diagnosis, number of dropouts, and the city and country where the intervention took place.

### Methodological quality assessment

Authors JB and JV-A assessed the methodological quality of the controlled studies using the Physiotherapy Evidence Database (PEDro) rating scale [47]. Any disagreement between the two reviewers about the methodological quality of the studies was resolved by consensus with the help of a third reviewer (GP-N). The PEDro scale consists of 11 items for evaluating the methodological quality of a study, although one of them (eligibility criteria item) does not contribute to the total score [47]. A score of 1 is obtained if the criterion is satisfied. Therefore, the PEDro total score ranges from 0 to 10, and the higher the score is, the better the methodological quality of the clinical trial. The PEDro scale was chosen based on its reliability, validity, and ease of use for evaluating randomized clinical trials and other controlled study designs in cognition-related interventions [48]. A PEDro score of 9–10 is considered “excellent,” 6–8 as “good”, and 4–5 as “fair”, whereas any score below 4 indicates a “poor” quality study [49].

## Results

### Data synthesis

**Fig. 1:**
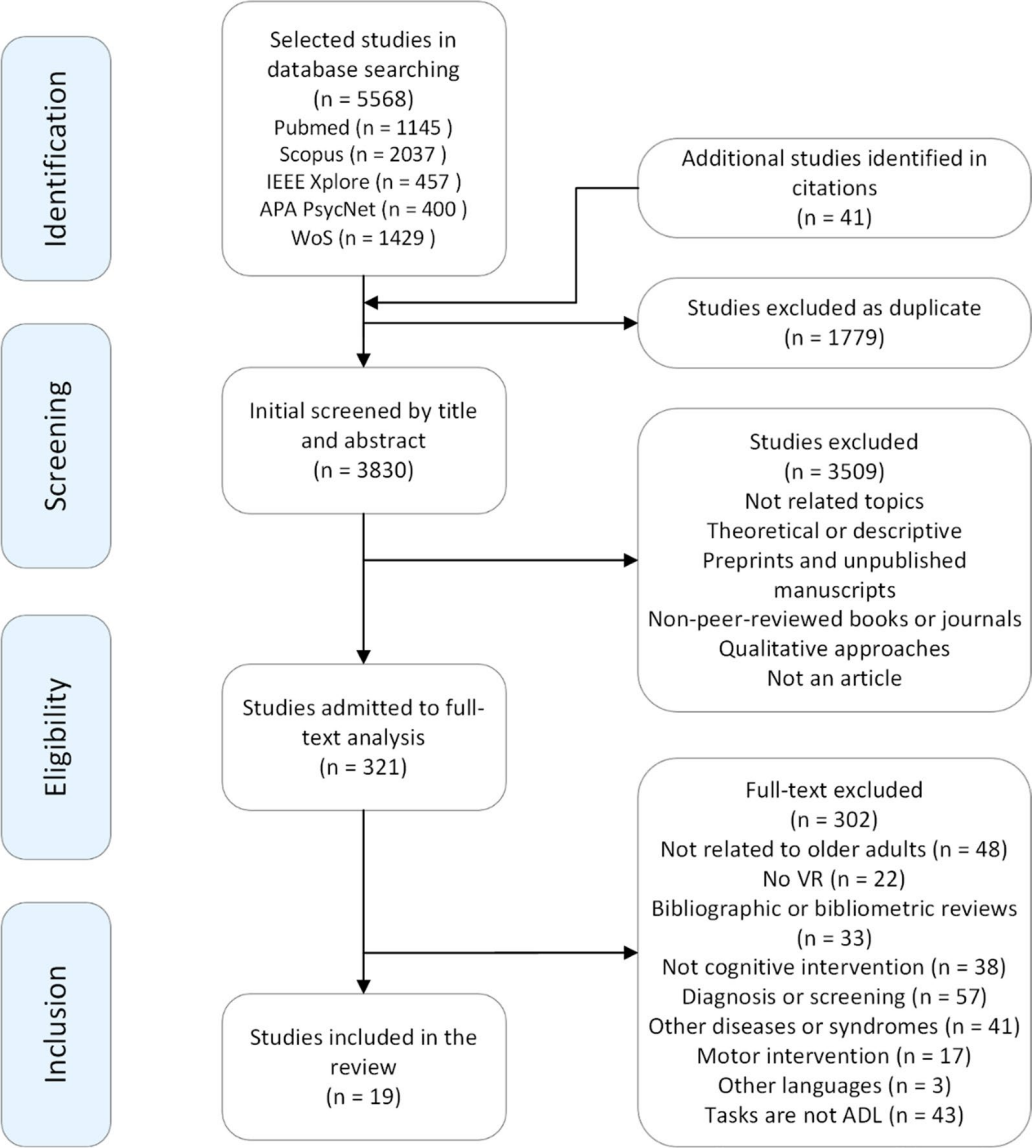
Consort diagram of study selection

The initial search yielded a total of 5568 articles from five databases. After removing 1779 duplicates, 3782 abstracts were further examined, and 3830 articles were excluded. Three hundred twenty-one articles were selected for full-text reading. After excluding 301 that met the eligibility criteria, 19 articles were included in the final review. The study selection process was conducted taking into account the PRISMA guidelines [50], and its results are summarized in Fig. 1.

### Methodological quality assessment

The PEDro scores for all controlled trials studies are presented in Table 1. They are not yet available in the PEDro database [47], so we had to evaluate them. Their final score was obtained by consensus between the authors. The twelve assessed studies ranged from 4 to 8 (out of 10), with an average score of 6.16 ± 1.14, which suggests that the included studies were of “good” methodological quality. The eligibility criteria were satisfied in all of the studies except that of Yamaguchi et al. [51]. All the studies included in our review satisfied the baseline comparability criteria. However, it is worth noting that, due to the nature of VR-based interventions, it can be challenging to apply some items of the PEDro scale, such as subject blinding and therapist blinding, as well as concealed allocation. This last item was not fulfilled in any of the studies we reviewed. Therefore, we considered studies with PEDro scores of 4 or higher to be of reasonable quality in our review [52]. However, it is important to acknowledge that applying concealed allocation and other criteria of the PEDro scale in VR intervention studies can be inherently challenging, and this should be considered when interpreting the results of our review and planning future research in this field.

**Table 1:** PEDro scores of the controlled-trial studies

Study	Eligibility criteria	Random allocation	Concealed allocation	Baseline comparability	Subject blinding	Therapist blinding	Assessor blinding	< 15% drop-out	Intention-to-treat analysis	Between group difference reported	Point estimate and variability reported	Total Pedro Score
Man et al. (2012) [59]	1	1	0	1	0	0	0	1	1	1	1	6/10
Kang et al. (2021) [61]	1	1	0	1	0	0	1	1	0	1	1	6/10
Schreiber et al. (1999) [58]	1	1	0	1	0	0	1	1	1	1	1	7/10
Panerai et al. (2021) [64]	1	0	0	1	0	0	1	1	1	1	1	6/10
Hofmann et al. (2003) [69]	1	0	0	1	0	0	0	1	1	1	1	5/10
Yamaguchi et al. (2012) [51]	0	0	0	1	0	0	0	1	1	1	0	4/10
Gamito et al. (2020) [56]	1	1	0	1	1	1	0	1	1	1	1	8/10
Park et al. (2019) [63]	1	1	0	1	0	0	1	1	1	1	1	7/10
Park (2022) [62]	1	1	0	1	1	0	1	1	1	1	1	8/10
Optale et al. (2010) [70]	1	1	0	1	0	0	1	1	0	1	1	6/10
Tarnanas et al. (2014) [54]	1	1	0	1	0	1	1	1	0	0	1	6/10
Oliveira et al. (2021) [60]	1	1	0	1	0	0	0	1	0	1	1	5/10
Studies’ degree of compliance (%)	91.16	75	0	100	16.66	16.66	58.33	100	66.66	91.16	91.16	

A score of 1 indicates that the study satisfied a specific criterion, while a score of 0 indicates that the study did not meet that criterion. It is important to note that the eligibility criteria item is not included in the calculation of the total PEDro score. The degree of compliance with the criteria of the different studies is expressed as a percentage of the total number of studies

### Overview of interventions

#### Participant characteristics

A total of 590 participants across nineteen studies were included in this review. The study of Foloppe et al. [53] considered only one person, whereas in Tarnanas et al.’s study [54], 105 people were included. The average number of participants per intervention was ≈ 31 (23). The age of the participants in all the studies was over 60 years, with an average age of 75.25 (5.55). A total of 165 men (28.01%) were included in the studies, on average ≈ 9 (9) per intervention and 424 women (71.99%), on average ≈ 24 (16) per intervention. Regarding the years of formal education, five studies did not report any information [54–58]. Man et al. [59] made a classification by years of study (0, 1–2, > 2) and Oliveira et al. [60] classified people according to their educational level. In the rest (n = 12), there was an average of 8.5 (2.05) years of formal education.

Concerning the cognitive diagnosis, patients with MCI (n = 4) were included [54, 61–63]; AD or dementia (n = 6) [51, 55, 57, 58, 60, 64]; healthy (n = 2) [56, 65]; MCI and healthy (n = 2) [66, 67]; MCI and mild dementia (MD) (n = 1) [68]; probable AD (pAD) (n = 2) [53, 59]; AD, depression and healthy (n = 1) [69] and MCI and memory deficit (n = 1) [70]. In (n = 8) [54, 57, 60, 61, 66–68, 70], dropouts were reported. Table 2 summarizes the demographic characteristics of the participants for each of the included studies. For each study, the following are shown: the authors and year of publication, the size of the sample and that of the experimental and/or control groups, the participant diagnosis, age, gender, years of formal education, number of dropouts, and the city and country where the intervention took place.

**Table 2:** Characteristics of the participants of the included studies

Authors and year of publication	Sample (n)	Diagnosis	Mean Age (Years) (SD)	Male/Female	Years of study (Years) (SD)	Dropouts	City, Country
Foloppe et al. (2018) [53]	1	pAD	79	0/1	6	DNM	Angers, France
Schreiber et al. (1999) [58]	14 (EG: 7; ACG: 7)	AD	EG: 80.86 (4.6); ACG: 78.86 (6.72)	EG: 2/5; ACG: 1/6	DNM	DNM	Düsseldorf, Germany
Zhu et al. (2022) [68]	31 (MCI: 18; MD: 13)	MCI, MD	MCI: 82.94 (6.44); MD: 85.76 (4.67)	MCI: 6/12; MD: 3/10	MCI: 11.00 (3.97); MD: 11.23 (4.71)	4 (MD: 1; MCI: 3)	Hong Kong, China
Man et al. (2012) [59]	44 (EG: 20; ACG: 24)	pAD	EG: 80.30 (1.21); ACG: 80.28 (1.31)	EG: 3/17; ACG: 2/22	EG: Un: 16; 1 year: 2; more: 2; ACG: Un: 14; 1 year: 4; more: 6	DNM	Hong Kong, China
Panerai et al. (2021) [64]	42 (EG. 24; AGC: 18)	AD	EG: 68.5; ACG: 64.0	EG: 13/11; ACG: 6/12	EG: 8; ACG: 8	DNM	Troina, Italy
Masoumzadeh et al. (2020) [57]	10 (H: 2; MCI: 4; AD: 4)	Dt	H: 85 (6); MCI: 79 (10.5); AD: 69 (7.1)	3/7	DNM	1 (H)	Winnipeg, Canada
Fasilis et al. (2018) [55]	10	Dt	73.6	DNM	DNM	DNM	Athens, Greek
Hofmann et al. (2003) [69]	28 (AD: 9; D: 9; H: 10)	AD, Dp	AD: 68.1 (14.7); D: 67.3 (9.4); H: 69.3 (5.8)	AD: 2/7; D: 2/7; H: 3/7	AD: 11.9 (5.4); D: 12.0 (5.7); H: 12.1 (5.2)	DNM	Basel, Switzerland
Kang et al. (2021) [61]	41 (EG: 23; PCG: 18)	sCD, MCI	EG: 75.48 (4.67); PCG: 73.28 (6.96)	EG: 6/17; PCG: 6/12	EG: 7.7 (4.1); PCG: 8.56 (4.83)	4 (EG: 2; PCG: 2)	Incheon, South Korea
Yamaguchi et al. (2012) [51]	4 (AD: 2; H: 2)	AD	AD: 78.5; H: 82.5	AD: 0/2; H: 1/1	8	DNM	Angers, France
Park (2022) [62]	32 (EG: 16; PCG: 16)	MCI	EG: 72.3 (5.13); PCG: 70.9 (4.51)	EG: 9/7; PCG: 6/10	EG: 7.56 (3.93); PCG: 7.50 (2.89)	DNM	Asan, South Korea
Maeng et al. (2021) [66]	47 (MCI: 24; H: 23)	MCI, H	MCI: 73.2 (7.3); H: 71.6 (4.4)	MCI: 8/23; H: 3/22)	MCI: 9.5 (4.7); H: 8.9 (3.4)	9 (MCI: 7; H: 2)	Incheon, South Korea
Kim et al. (2021) [67]	44 (MCI: 22; H: 22)	MCI, H	MCI: 74.23 (7.5); H: 71.45 (3.95)	MCI: 5/17; H: 2/20	MCI: 8.68 (4.61); H: 8.91 (3.16)	11 (MCI: 9; H: 2)	Incheon, South Korea
Park et al. (2019) [63]	21 (EG: 10; ACG: 11)	MCI	EG: 70.5 (4.2); ACG: 72.6 (5.3)	EG: 2/8; ACG: 2/9	EG: 7.09 (3.36); ACG: 7.09 (3.36)	DNM	Daegu, South Korea
Gamito et al. (2019) [65]	25	H	74 (5.27)	4/21	6 (2.42)	DNM	Benfica, Portugal
Gamito et al. (2020) [56]	43	H	75 (5.43)	9/34	DNM	DNM	Lisbon, Portugal
Optale et al. (2010) [70]	31 (EG: 15; ACG: 16)	MCI y Mdf	EG: 78.5 (10.9); ACG: 81.6 (5)	EG: 5/10; ACG: 5/11	EG: 5.3 (2.4); ACG: 6 (3.5)	5 (EG: 3; ACG: 2)	Venetia, Italy
Tarnanas et al. (2014) [54]	105 (EG: 32; ACG: 39; PCG: 34)	MCI	EG: 70.5; ACG: 69.7; PGC: 70.9	EG: 12/20; ACG: 16/23; PCG: 13/21	DNM	9 (EG: 7; PCG: 2)	Kozani, Greek,
Oliveira et al. (2021) [60]	17 (EG: 10; PCG: 7)	AD	83.24 (5.66)	EG: 3/7; EG: 2/5	EG: Un: 2, Pri: 6, Sec or more: 2; CG: Un: 0, Pri: 5, Sec or more: 2	EG: 1	Lisbon, Portugal

H: Healthy, Dt: Dementia, pAD: Probable Alzheimer’s Disease, Dp: Depression, sCD: Subjective Cognitive Impairment, Un: Below than primary school, Pri: Primary education, DNM: Does not mention, Mdf: Memory deficit, SD: standard deviation, Sec: Secondary Education

#### Characteristics of interventions

Of the 19 studies in our review, one case study, six pilots, six clinical trials, and 6 randomized controlled trials (RCTs) were identified. The case study provided a detailed view of a specific case [53], while the pilots offered preliminary information on the effectiveness and feasibility of VR interventions in iADL [51, 55, 57, 58, 63, 68]. Clinical trials (CTs) evaluated the effectiveness in larger groups of participants [56, 64–67, 69], and the RCTs provided methodological rigor by randomly assigning participants to intervention and control groups [54, 59–62, 70].

In one study [62], a passive control group (PCG) was used. Seven studies [56, 58–61, 63, 70] used an active control group (ACG). Tarnanas et al. [54] used both a PCG and an ACG. Ten studies [51, 53, 55, 57, 64–69] involved only virtual therapy.

- *Intervention type.* Regarding the type of intervention, three groups were identified: cognitive training (CTR), cognitive rehabilitation (CRE), and cognitive stimulation (CST). Thirteen studies [57–59, 61–64, 66–70] presented a CTR intervention. Two studies [53, 55] described that their interventions are within the CRE. Four studies [54, 56, 60, 65] carried out a CST, and in [51], a relearning program was implemented. Eleven interventions [51, 53–55, 57, 58, 62, 66–68, 70] simulated a single task, while the others simulated multitasking.
- *Cognitive domain.* A total of 32 different cognitive domains were identified across the 19 studies included in this review. Of these, 12 studies focused on executive functions [54–56, 60–63, 65–68, 70], 10 on attention [53–56, 62, 63, 65–68], and 8 on visuospatial processing [54, 57, 61, 62, 64, 67, 69, 70]. Other domains evaluated included memory (n = 6) [53, 54, 66–69], iADLs (n = 6) [53, 56, 60, 62, 64, 70], general or global cognition (n = 5) [56, 60, 64, 68, 70], verbal memory (n = 4) [61, 63, 64, 70], and working memory (n = 4) [55, 56, 63, 65].
- *Simulated task.* VR applications that simulated iADLs were highly heterogeneous. Five studies [62, 66–69] simulated purchases in a store/supermarket. In two studies [58, 63], VR tasks were performed in an apartment. One study implemented kitchen tasks [51], and Foloppe et al. [53] alternated between a real kitchen and a virtual kitchen. A driving simulator was described in [57], a museum in [54], and auditory training in everyday places in [70]. There are also combined tasks (kitchen and apartment) [59], virtual supermarket, kitchen and apartment [55], and various tasks (including supermarket and kitchen tasks) in five studies [56, 60, 61, 64, 65].
- *Level of immersion.* Regarding the levels of immersion with which VR is applied, seven studies [57, 61, 63, 66–68, 70] used immersive environments, while in the rest of the studies, tasks were performed in non-immersive environments [51, 53–56, 58–60, 62, 64, 65, 69]. One of the negative effects that immersive virtual environments can produce is cybersickness, which includes dizziness, disorientation, headaches, eye pain, and other related pain. Four studies [57, 61, 66, 68] reported the presence of these symptoms (mostly mild).
- *Number of sessions and duration.* The average number of sessions was 17.6 (14.74), with a minimum of 2 [51] and a maximum of 60 [70]. One intervention [64] was conducted in group sessions (with 5 participants), and in the study of Park [62], it is unclear whether it was individual or group-based. The duration of each session was not mentioned in the study of Hofmann et al. [69], resulting in an average session time of 12.81 (13.79) hours per person on average. Regarding the type of VR applied, the duration of the sessions in interventions with immersive VR ranged from 20/30 min to 50/60 min. Interventions with non-immersive VR had a duration ranging between 30 and 90 min. The interventions ranged from a minimum of 3 [51] and 4 [61] hours, to a maximum of 60 h [54]. Interventions lasting less than 10 h were proposed in eleven studies [51, 56–61, 63, 65–67], while four studies [53, 55, 62, 64] used between 10 and 19 h, and three studies [54, 68, 70] used 20 h or more. Medication use (pharmacotherapy) during the intervention was reported in three studies [61, 64, 69]. Follow-up or maintenance evaluations were not performed in 18 studies, while in [53], two follow-up assessments were conducted (at one month and six months later), and Hofmann et al. [69] conducted a follow-up after three weeks.
- *Administrators of the interventions.* Occupational therapists administered the intervention in eight studies [51, 53, 58, 59, 62, 63, 66, 70]. In five studies, the treatment was administered by a clinical psychologist [54, 56, 64, 65, 68], whereas in two studies, a clinical neuropsychologist was responsible [60, 61] Finally, four studies did not mention who conducted the intervention [55, 57, 67, 69].
- *Country.* South Korea had the most interventions (n = 5) [61–63, 66, 67], followed by Portugal (n = 3) [56, 60, 65]. Other countries, such as France [51, 53], Greece [54, 55], Italy [64, 70] and China [59, 68], contributed two studies each. Canada [57], Germany [58] and Switzerland [69] contributed one study each.

Table 3 shows the main characteristics of the interventions, including who administered the interventions, the design, type of intervention and level of immersion, virtually simulated place, duration of treatment, outcome measurements, main findings, and conclusions. Since some tasks (e.g. supermarket) can include different subtasks, we also include in Table 4 all the subtasks corresponding to these high-level activities.

**Table 3:** Characteristics of the interventions in the included studies

Authors and year of publication	Study type	Intervention and VR type	Task/place simulated	Duration (Days × weeks × minutes)	Outcome measurements	Specific cognitive abilities	Main findings	Conclusions
Foloppe et al. (2018) [53]	Single case	Non-Immersive CRE	Virtual and real cooking tasks	4d × 4w × 60 min	Patient: MMSE, IADL-9, RDES, ADRQL-F. Caregiver: MiniZarit, RSES, ADRQL-F	Memory, attention, planning, sequencing, basic motor skills, iADL	The patient showed a modest but significant improvement in assisted autonomy when relearning cooking activities with VR (15.4% in the virtual condition, 11.3% in the real condition) The need for assistance decreased, although instrumental activities of daily living did not change Mini-Zarit (caregiver burden) was reduced from moderate to mild	VR enabled the AD patient to improve in cooking tasks, with stable results up to a 6-month follow-up. However, there was no knowledge transfer to other iADLs Hybrid programs combining VR and real environments may be beneficial in achieving generalization to other activities Therefore, VR interventions should be tailored to individual needs and histories
Schreiber et al. (1999) [58]	Pilot pretest–posttest	Non-Immersive CTR	Virtual apartment	5d × 2 w × 30 min	NAI Picture Test, NAI Figure Test, RBMT Picture Test, RBMT Route Learning (immediate and delayed)	Immediate and late retention and visual-figurative and topographic material	VR training improved the retention of significant visual-figurative and topographic information, with notable differences between the training and control groups on the NAI Picture Test (P = 0.006) and the RBMT Route Learning, delayed (P = 0.025) Subjects with dementia experienced real improvements in training and achieved lower difficulty levels on delayed retention	Pre- and post-training neuropsychological assessment indicated a direct transfer to real-life situations in the training group Standardized psychometric tests revealed a positive effect of computer-based training in certain memory domains, specifically in the target areas
Zhu et al. (2022) [68]	Pilot pretest–posttest	Immersive CTR	Virtual Supermarket	3 d × 5 w × 20/30 min	MoCA-CS, MMSE, AVLT, STT, SDMT, PSS, GDS	Executive function, attention, memory, general cognition	Both groups (MCI and MD) showed significant improvements in all measures of cognitive function The MD group improved overall cognitive function significantly more than the MCI group in MoCA, SDMT, STT, and AVLT In addition, an intervention effect was found in improving perceived stress	VR-based intervention protocol was effective in improving the general cognitive function (memory, executive function, and attention) of patients with MCI and MD Adjusting task difficulty according to the baseline cognitive level of patients with MD positively improved cognitive function
Man et al. (2012) [59]	RCT	Non-Immersive CTR	Virtual apartment, Virtual supermarket	2/3 d × 4 w × 30 min	FOME-TE, FOME-TR, FOME-DR, MMQ-contentment, MMQ-ability, MMQ-strategy, HK Lawton IADL	episodic memory, total recall, delayed recall, memory, satisfaction with performance, iADL	The VR group showed significant improvements in total encoding, total recall and delayed recall (FOME), and MMQ strategy The active control group improved total recall, delayed recall (FOME), and MMQ satisfaction	Virtual training significantly improved memory performance (immediate and delayed episodic memory recall) regardless of educational level VR environments with audiovisual stimuli can improve information encoding
Panerai et al. (2021) [64]	CT	Non-Immersive CTR	1) Follow instructions 2) Medication intake 3) Fill a suitcase 4) Virtual supermarket	5d × 6w × 3 h (5-people group)	MMSE, CPM, CBT, DS, RAVL (immediate, delayed), FAB, IADL	General cognition, ecological memory, visual-auditory memory, selective attention,verbal memory, iADL	The experimental group (EG) showed improvements in correct responses, missing responses, number of cues and execution times compared to the active control group (ACG) There was no improvement in the iADL scale in both groups A spontaneous transfer of re-learned functional skills to iADL in EG is suggested	VR showed ecological validity, improving functional skills in patients with early-stage dementia Daily living skills need specific training to be relearned Skill generalization is related to cognitive functioning and hippocampal integrity
Masoumzadeh et al. (2020) [57]	Pilot pretest–posttest	Immersive CTR	Driving simulator (VRDS)	5d × 4w × 30 min	MWT, SSQ, MADRS	spatial cognition, independence, mood, depression	Significant advances in spatial cognition (Morris test) and learning in older adults with varying degrees of dementia Improvements in mood and cognitive functions More training time is suggested for patients with MCI and AD	VRDS training had positive effects on older adults’ spatial cognition, even on those with different degrees of dementia There were also improvements in mood and cognitive functions
Fasilis et al. (2018) [55]	Pilot pretest–posttest	Non-Immersive CRE	Virtual supermarket, Virtual cooking tasks, home arrangement and cleaning	3d × 5w × 40 min	DS, BSRT, TMT-A, Hanoi Tower, FAB, WCST	Working memory, attention, problem-solving, rigid thinking, executive function	Significant improvements in working memory, memory retention, executive functions, and rigid thinking Statistically significant increase in FAB battery (executive functions) between pre-and post-training Knowledge transfer to daily activities observed	Relative improvement in the total cognitive variables considered Possible ceiling effect in healthy participants. Therapeutic benefits and transferability to real-life situations in 3D virtual environments Need for further research to assess the "therapeutic effect"
Hofmann et al. (2003) [69]	CT	Non-Immersive CTR	Virtual Supermarket	3d × 4w	CDR, MMSE, TMT-A, MADRS	Navigation, decision making, memory, basic motor skills	Improvement trend in all three groups, but only the reduction in errors in patients with AD was significant Improvements in all groups up to 3 weeks later Patients with depression presented higher latency than healthy patients, and no correlation was found between MADRS and performance	The intervention showed promising results, especially in reducing errors in patients with AD However, further research is needed to address methodological limitations and assess the ecological validity of computer-based ADL applications in different patient groups
Kang et al. (2021) [61]	RCT	Immersive CTR	Find differences, select objects to perform tasks, Virtual supermarket, find a path, place furniture in a space, catch animals in a certain order	2d × 4w × 20/30 min	ROCF, MMSE, DS, TMT-A, K-BNT, SVLT, COWAT, SCWT, TMT-B, GDS, AES, PANAS-P, PANAS-N, QoL-AD	Visuospatial processing, naming ability, verbal memory, phonetic fluency, frontal executive function and psychiatric symptoms	The intervention showed significant improvements in the EG in the total score and basic components of the RCFT Non-significant improvements were found in naming skills, delayed verbal memory recall, and phonetic fluency Improvements in psychiatric symptoms such as apathy, affect, and quality of life in the EG	The intervention positively affected various cognitive functions and psychiatric conditions, as well as increased frontal-occipital functional connectivity in older people in the predementia state Future research with larger samples and control groups is needed to validate these results
Yamaguchi et al. (2012) [51]	Pilot pretest–posttest	Non-Immersive Re-learning	Virtual cooking tasks	2d × 1w × 90 min	Written ILM, Self-recorded ILM	Ability to learn and perform ADL in virtual environments	Patients with AD take longer in daily functional activities. Error-free learning methods in virtual environments could benefit patients with AD Participants reported difficulties with mouse use and needed more time to become familiar with virtual tasks	Training in virtual environments may increase functional autonomy in patients with AD However, further studies are required to confirm these results, considering the limitations, such as the low number of participants, differences in AD stages, and few training sessions
Park (2022) [62]	RCT	Non-Immersive CTR	Virtual supermarket	2d × 8w × 60 min	EFPT-K, K-IADL	Visual memory, attention, navigation, executive function, iADL	The study found that virtual shopping training significantly improved executive functions and iADLs in patients with MCI compared to the control group, supporting the ecological validity of this approach	Virtual shopping training is clinically effective in improving executive function and iADLs in patients with MCI Shopping in virtual environments can be an effective form of training, as it requires complex cognitive skills and helps patients cope with distractions
Maeng et al. (2021) [66]	CT	Immersive CTR	Virtual supermarket	2d × 4w × 50/60 min	CERAD-K, KQOL-AD, GDS, SSQ, Presence Q, Presence S	Memory, attention, executive function	Improvements in cognition in patients with MCI and healthy older adults Both groups improved in learning new information, visuospatial construction, and frontal lobe function The MCI group specifically improved in word recall and recognition and TMT-A performance, while the healthy older adult group improved on the Korean-Boston naming test	VRCT improves cognition in patients with MCI and healthy older adults Despite limitations, such as a small sample size and lack of follow-up, the study suggests that VRCT programs may be applicable and effective in populations with reduced cognitive ability
Kim et al. (2021) [67]	CT	Immersive CTR	Virtual supermarket	2d × 4w × 50/60 min	CERAD neuropsychological battery, CRI (total, education, working activity, leisure time)	Memory, attention, executive function, verbal memory, visuo-spatial processing, learning capacity	Greater improvement in the total CERAD score was found for cognitively normal participants with higher versus lower scores on the Education subdomain of the CRIq Healthy older adults and patients with MCI improved on CERAD tests after using a cognitive training application Education was associated with better CERAD scores, whereas leisure time showed a negative correlation	The results suggest that cognitive training benefits healthy older adults and patients with MCI Education is an important factor in cognitive rehabilitation, while leisure time and age also influence outcomes Sex showed no relationship with the results
Park et al. (2019) [63]	Pilot pretest–posttest	Immersive CTR	Virtual apartment	3d × 6w × 30 min	CERAD-K, TMT-A, TMT-B, CDR, MMSE, BDI, MBI	Attention, visual-auditory memory, verbal memory, executive function	Significant improvement in the experimental group in the constructive recall test, which measures visuospatial working memory and recalls in visuospatial tasks There were no significant interactions in the other tests	Mixed Reality-based cognitive training may benefit visuospatial working memory in patients with MCI Further research with larger samples, longer sessions, and longer follow-ups is required
Gamito et al. (2019) [65]	CT	Non-Immersive CST	Virtual cooking tasks, remembering TV news, choosing clothes and shoes arrangement	2d × 6w × 30 min	MoCA, FAB, WCST, RCFT, d2, ECQ, BDI- II	visual-auditory memory, attention, cognitive flexibility, executive function, working memory	Statistically significant improvements in attention (d2), in visual memory (RCFT) and in two indicators of cognitive flexibility (WCST) The magnitude of improvements is highest for individuals with lower levels of cognitive functioning at baseline No effects of age or education were found	Attention improved more in people with greater impairment, making the system more efficient for training patients with signs of cognitive decline The specific functions in which significant differences were observed (attention, memory, and cognitive flexibility) are required in daily activities
Gamito et al. (2020) [56]	CT	Non-Immersive CST	Personal hygiene, virtual cooking tasks, virtual supermarket, tasks related to TV news, clothes, shoes, art gallery visit)	EG: 2d × 6w × 30 min y ACG: 1d × 6w × 60 min	MoCA, FAB, RCFT, d2, WMS-R, GDS, SWLS, IADL	General cognition, attention, concentration, executive function, working memory, iADL	Significant improvements in EG in global cognition (14%), EF (13%), memory (WMS-R), attention (d2), and visual memory (RCF) There were no improvements in functionality or subjective well-being Results in well-being and functionality were not significant in any of the groups	The virtual SLB environment is more effective than traditional methods in improving general cognition, attention, memory, and EF in older adults No improvements in well-being or functionality were observed Further studies are needed to assess the effects on well-being and functioning in cognitively impaired adults
Optale et al. (2010) [70]	RCT	Immersive CTR	Auditory and VR experience sessions in everyday environments	TR: 3d × 12w × 30 min; reinforcement: 2d × 12w × 30 min	MMSE, MS, DS, VSR, PVF, DTP, CET, CDT; ADL-F, ADL-M, GDS, IADL	General cognition, verbal memory, executive function, visual-auditory memory, visuospatial processing, depression, iADL	Improvements in the EG in general cognitive skills, verbal memory, and executive functions with VRMT; the CG had decreases in the same areas The booster sessions consolidated the effects of the training (MMSE, short-term and long-term memory, and DTP) with small effects No differences were found in visuospatial processing, iADL, or GDS	VRMT showed positive effects on long-term memory and stimulated cognitive abilities Improvements in executive functions soon faded, and there were no improvements in visuospatial skills
Tarnanas et al. (2014) [54]	RCT	Non-Immersive CST	Virtual Museum	2d × 20w × 90 min	RAVLT, ROCF, DS, SCWT, TMT-A, TMT-B, BNT, MMSE, GDS, Category fluency, Letter fluency	Memory, attention, executive function, navigation, visuospatial processing	After five months of intervention, there were significant improvements in the RAVLT, King Test, Trail-making B, and MMSE tests in the experimental group (EG) There was also an improvement in the GDS scale, although not statistically significant, and an overall improvement in the participants’ scores	The training proved effective in improving visual arrays’ textual and spatial processing. This approach also showed improvements in untrained tasks and the perceived cognitive performance of older adults with aMCI in everyday life However, it is suggested that physical exercise interventions may be more effective in addressing executive function impairment in this population
Oliveira et al. (2021) [60]	RCT	Non-Immersive CST	Personal hygiene, virtual cooking tasks, virtual supermarket, tasks related to TV news, clothes, shoes, art gallery visit	2d × 5w × 45 min	FAB, MMSE, IADL, TMT-A, TMT-B, CDT, GDS, CDR	Executive function, general cognition, depression, iADL	Increase in mean FAB score and a significant and large effect on global cognition between pre- and post-treatment assessments However, no improvements in executive functions iADL were found	Mixed effects on cognitive function when using VR as an intervention Despite the lack of improvements in specific cognitive functions, an improvement in global cognitive functioning was observed Cognitive reserve (CR) theory could explain individual differences in the ability to maintain cognitive function in the presence of brain pathology

ADL-F: Activities of Daily Living–Functions; ADL-M: Activities of Daily Living–Mobility; ADRQL-F: Alzheimer’s Disease-Related Quality of Life—French version; AES: Apathy Evaluation Scale; aMCI: amnestic Mild Cognitive Impairment; AVLT: Auditory Verbal Learning Test; BDI: Beck Depression Inventory; BNT (K-): Boston Naming Test (Korean version); CBT: Corsi Block-tapping Task; CDR: Clinical Dementia Rating; CDT: Clock Drawing Test; CERAD (-K): Consortium to Establish a Registry for Alzheimer’s Disease (Korean version); CET: Cognitive Estimation Test; CPM: Colored Progressive Matrices; CRIq: Cognitive Reserve Index questionnaire; DNR = Does not report; DS: Digit Span Test; DTP: Dual Task Performance; d2: d2 Test of Attention; ECQ: Everyday Competence Questionnaire; EFPT(-K): Executive Function Performance Test (Korean version); FAB: Frontal Assessment Battery; FOME (‐TE, -TR, -DR): Fuld Object Memory Evaluation (-total encoding, -total retrieval, -delayed recall); GDS: Geriatric Depression Scale; IADL-9; Instrumental Activities of Daily Living Scale – 9 items; ILM: Instruction Learning Methods; Lawton IADL (HK, K-): Lawton Instrumental Activities of Daily Living scale (Hong Kong Chinese version, Korean version); MADRS: Montgomery-Asberg Depression Rating Scale; MBI: Modified Barthel Index; MMQ: Multifactorial Memory Questionnaire; MMSE: Mini-Mental State Examination; MoCA (-CS): Montreal Cognitive Assessment Scale (Chinese-Changsha version); MS: Mental Status in Neurology; MWT: VR Morris water test; NAI: Nuremberg Aging Inventory; PANAS-N: Positive and Negative Affect Schedule-negative affect; PANAS-P: Positive and Negative Affect Schedule-positive affect; Presence Q: Measuring presence of VR contents; Presence S: Measuring presence of VR content users; PSS: Perceived Stress Scale; P300: Peak of the Event-related-potencial occurring at a latency of 300 ms.; QoL-AD (K): Quality of Life-Alzheimer’s Disease (Korean version); RAVLT: Rey Auditory Verbal Learning Test; RBMT: Rivermead Behavioral Memory Test; RCFT: Rey Complex Figure Test; RE = Cognitive rehabilitation; ROCF: Rey–Osterrieth complex figure test; RSES: Rosenberg Self-Esteem Scale; SBL: Systemic Lisbon Battery; SCT: Stroop Color Test; SCWT: Stroop Color and Word Test; SDMT: Symbol Digit Modalities Test; SSQ: Simulator Sickness Questionnaire; ST = Cognitive stimulation; STT: Shape Trail Test; SVLT: Seoul Verbal Learning Test; SWLS: Satisfaction with Life Scale; TMT-A: Trail Making Test Part A; TMT-B: Trail Making Test Part B; TR = Cognitive training; VD = Virtual department; VS = Virtual supermarket; VSR: Verbal Story Recall Test; WCST: Wisconsin Card Sorting Test; WMS-R: Wechsler Memory Scale-Revised

**Table 4:** Description of the subtasks for each study

Author	Task	Subtasks
Foloppe et al. (2018) [53]	Virtual and real cooking tasks	10 different tasks. Namely, setting the table for two people, making a cup of coffee with a coffee machine, making a cup of coffee with an espresso machine, making a cup of sweet tea with a kettle, making breakfast, making a salad with three ingredients, heating soup in a microwave, preparing frozen vegetables with an electric or induction stove, making a sandwich, and cooking a chocolate cake with an electric oven
Schreiber et al. (1999) [58]	Virtual apartment	The virtual apartment consisted of five rooms, one of which was a corridor connected to all the other rooms. In stimulus set I, a room was chosen and subjects were asked to find certain targets. In stimulus set II, subjects were asked to find certain rooms in the apartment
Zhu et al. (2022) [68]	Virtual Supermarket	A list of 3 to 12 familiar virtual images and labels of common items (such as oranges or toothpaste) were showed to participants. After that, they were asked to memorize them in one and a half minutes. After memorizing the list, the participants played a number sorting game for 20 s before going to the supermarket to buy the items on the memorized list. Participants were then asked to locate the counter and pay in RMB bills
Man et al. (2012) [59]	Virtual apartment, convenient shop	Virtual apartment. The 3-min task involved moving around the apartment, reading and memorizing the items on a list placed on a table. They then took those items out of the refrigerator, after a period of distraction Virtual convenient shop. Participants were asked to search at the store and purchase the requested items. After that, they had to pay their bills to the cashier
Panerai et al. (2021) [64]	1) Follow instructions 2) Medication intake 3) Fill a suitcase 4) Virtual supermarket	1) Following instructions. Participants were asked to answer 30 questions of general knowledge, personal, family, spatial and temporal orientation, which appeared on the screen in verbal and written form 2) Medication. Five boxes of medicines placed on a kitchen table, verbal instructions and a visual reminder were presented. The subject had to indicate the correct time and the respective medication 3) Fill a suitcase. The subject was required to pack a suitcase for a weekend. The subject dragged the clothes from a shelf to the suitcase 4) Supermarket: A shopping list was displayed on the screen and items were dragged from the shelf to the cart. To do the payment, the participant had to drag the bills from the wallet
Masoumzadeh et al. (2020) [57]	Driving simulator (VRDS)	The user is asked to follow a path. The simulator recorded whenever the participant stopped with a red light, a stop sign and whenever they turned to the correct directions at intersections
Fasilis et al. (2018) [55]	Virtual supermarket, Virtual cooking tasks, home arrangement and cleaning	The first task was based on the activity of buying in a supermarket. The second task dealt with the preparation of breakfast, and the third task required the patient to fix and clean his/her house. (No more information was offered)
Hofmann et al. (2003) [69]	Virtual Supermarket	Participants were asked to learn a shopping list of three items. The correct item had to be freely recalled and/or chosen from a multiple-choice list. Also, 10 multiple-choice questions related to the environment had to be answered
Kang et al. (2021) [61]	Find differences, select objects to perform tasks, Virtual supermarket, find a path, place furniture in a space, catch animals in a certain order	(i) Find differences), (ii) select items needed to perform certain tasks, (iii) prepare an exact amount of money to pay, (iv) find a way using a memorized map, (v) place furniture in space exactly based on a memorized drawing, (vi) remember words, (vii) remember flags and symbols, and (viii) catch animals in a certain order
Yamaguchi et al. (2012) [51]	Virtual cooking tasks	Breakfast task. To place two toasts in an electric toaster, press a lever, place the toasts on two different plates, put butter and jam on each bread Coffee task. To open the coffee pot, put the filter and put coffee, fill the pot with water, put the coffee pot on the heater and turn on. Serve the coffee in a cup, add sugar and milk
Park (2022) [62]	Virtual supermarket	A list of purchases was showed on the screen and the user selects them by touching the screen. Then the participant had go to the cashier and pay by choosing the precise amount of money
Maeng et al. (2021) [66]	Virtual supermarket	A list of items was given for 10 s at the entrance of the supermarket and then participants visited seven sections. Participants could use a hint button to display the list for 10 more seconds
Kim et al. (2021) [67]	Virtual supermarket	A list of items was given for 10 s at the entrance of the supermarket and then participants visited seven sections. Participants could use a hint button to display the list for 10 more seconds
Park et al. (2019) [63]	Virtual apartment	Living room: to turn on a child’s favorite cartoon TV channel, to make a phone call, and to play a game of cards Kid’s room: to make a kid clean his room, to choose books to read to a child, to match picture blocks, to pack a child’s school bag, to see shopping items, to color, to hang clothes and to play a matching game of colors Cooking: make sandwiches and prepare a bowl of cereals Toilet: to brush a child’s teeth and to teach him to use the toilet
Gamito et al. (2019) [65]	Virtual cooking tasks, remembering TV news, choosing clothes and shoes arrangement	Morning hygiene: to brush teeth Shoes arrangement: to put the shoes in order Wardrobe: to combine clothes to get dressed Virtual kitchen: to choose the ingredients from a list that is displayed on the screen Newscast: to turn on the TV and change the channels until finding the news channel. Remember the news Supermarket: to look at a shopping list, choose the items and pay. Pharmacy: to look at a shopping list, choose the items and pay Art Gallery: to browse the art gallery viewing the paintings Outdoors: to walk through the city and cross the streets carefully
Gamito et al. (2020) [56]	Personal hygiene, virtual cooking tasks, virtual supermarket, tasks related to TV news, clothes, shoes, art gallery visit)	Morning hygiene: to brush teeth Shoes arrangement: to put the shoes in order Wardrobe: to combine clothes to get dressed Virtual kitchen: to choose the ingredients from a list that is displayed on the screen Newscast: to turn on the TV and change the channels until finding the news channel. Remember the news Supermarket: to look at a shopping list, choose the items and pay. Pharmacy: to look at a shopping list, choose the items and pay Art Gallery: to browse the art gallery viewing the paintings Outdoors: to walk through the city and cross the streets carefully
Optale et al. (2010) [70]	Auditory and VR experience sessions in everyday environments	Listening sessions in familiar settings, such as the family home from childhood or a park. The participant had to remember different paths that were shown. Afterwards, the participant had to walk through them without losing orientation. These paths were individualized with color or shape indicators
Tarnanas et al. (2014) [54]	Virtual Museum	Listen and Plan. Participants followed instructions to locate and find items in the museum Storytelling. Participants listened to story segments of museum items and answer a series of questions Exercise games. Participants developed a "scene" based on archaeological artifacts or their multimedia description
Oliveira et al. (2021) [60]	Personal hygiene, virtual cooking tasks, virtual supermarket, tasks related to TV news, clothes, shoes, art gallery visit	Morning hygiene: to brush teeth Shoes arrangement: to put the shoes in order Wardrobe: to combine clothes to get dressed Virtual kitchen: to choose the ingredients from a list that is displayed on the screen Newscast: to turn on the TV and change the channels until finding the news channel. Remember the news Supermarket: to look at a shopping list, choose the items and pay. Pharmacy: to look at a shopping list, choose the items and pay Art Gallery: to browse the art gallery viewing the paintings Outdoors: to walk through the city and cross the streets carefully

## Discussion

This systematic review focused on evaluating the use of VR as a therapy in cognitively healthy older adults and those with cognitive disorders, specifically, MCI and different types of dementia, which are degenerative processes that gradually evolve over time. The reason for including healthy older adults was to provide a comprehensive perspective of the impact of virtual reality on both normal and pathological cognitive aging. This review brings important evidence about iADL-VR applications that report improvement in the cognitive domains involved in these activities and even in motivational and behavioral aspects. Previous studies have already reported moderate to large improvements in global cognition, memory, and executive function in VR interventions [31]. Furthermore, VR has been found to promote the reactivation of some areas of the cortex by boosting the processes of neuroplasticity, language, executive function, short-term and working memory, attention, movement, and balance [28]. According to Vallejo et al. [71], studies that also simulate everyday tasks could have a greater effect on executive functions, prospective memory, and retrospective memory.

### Cognitive improvement

Virtual environments that simulate activities of iADL can increase ecological validity and have positive effects on general cognitive function [68] and learning new information [66]. In the context of aging, tasks related to shopping maintain and improve independent daily functioning, planning, and problem-solving abilities [45]. These tasks are more complex and require participants to plan, organize, problem-solving, and multitask in a spatial and visual context, making them particularly relevant for autonomous daily living [72]. The review included studies that reported shopping, food preparation, and cleaning as the most common iADL performed by older adults. However, some complex iADLs were not considered in the interventions, possibly because they require more skill or resources [73]. Gamito et al. [56] simulated several ADLs and found a 14% improvement in global cognition in the intervention group, while Foloppe et al. [53] reported greater autonomy and reduced need for instructions.

Increased retention of visual-figurative material [58], general memory [68], visuospatial memory [56, 57, 63] and working memory [57, 63] has been identified. Additionally, improvements have been observed in executive functions [57, 68], retention, rigid thinking [55], cognitive flexibility [66], attention [68] and specifically selective attention (shopping list in a virtual supermarket) [66]. Improvements have also been reported in total and episodic encoding, total and directed recall [59, 61], constructive recall [63], verbal learning [57, 70] and phonetic fluency [61]. With regard to the transfer of knowledge to real life and even to other ADLs, several scales have been used, such as the Lawton scale [74], which was the most commonly used in the reviewed studies. It is worth mentioning that this scale uses a questionnaire rather than performance observation, which could lead to overestimation or underestimation of a skill. However, the cognitive domains developed in the different interventions are necessary for the functional development of ADLs. For example, the cognitive, visual-perceptual processing, and attention that were developed in simulated household tasks in the work of Fasilis et al. [55], or the improvements in cognitive flexibility, attention, and memory achieved by participants in the work of Gamito et al. [63]. A recent meta-analysis concluded that cognitive training achieves little transfer to functions that have not been trained [75].

Regarding performance on the different memory tasks included in the review, most research emphasizes that cognitively healthy individuals perform better [27, 76], followed by those with mild cognitive impairment [30, 77, 78], due to the typical learning difficulties associated with the disease [64]. For example, in virtual supermarket tasks, it was shown that patients with AD have worse performance and longer execution times [69], which are frequent in patients with neurological problems when executing ADLs [79]. In line with the meta-analysis of Kim et al. [80], the positive effects of VR are more noticeable in people with cognitive impairments. It has also been mentioned that VR could promote the activation of the intuitive system and the transfer of knowledge to another context but in the early stages of Alzheimer’s disease [64]. This was also confirmed by Hofmann et al. [69], where AD patients reduced errors as the training program progressed. It is important to note that the effectiveness ceiling may be a factor to consider in analyzing the results, especially in improving specific cognitive tasks already at high levels in the target population. Further studies are needed to assess whether there are significant differences in cognitive improvement between healthy individuals and those diagnosed with a cognitive disorder.

### Level of immersion in VR

The level of immersion in VR interventions for memory training remains a topic of debate. In our review, almost half of the interventions were immersive, and almost all were developed within the last four years. This contrasts with the review conducted by Kim et al. in 2019, which included only one immersive study [80]. The authors concluded that semi-immersive technology was more effective than fully immersive technology based on the effect sizes of the different studies. However, the low presence of interventions with immersive systems makes it difficult to generalize these findings to the present day, where the technology is further developed. Regarding performance in memory tasks, studies such as that of Krokos et al. [81] and Huttner et al. [82] reported better results using immersive environments compared to non-immersive ones. However, both Maidenbaum et al. [83] and Varela-Aldás et al. [9] found no significant differences in spatial memory performance between using a standard computer screen and a VR device (HMD). These results indicate that, to date, findings are inconclusive; therefore, there is a need for future research in this constantly evolving field.

VR immersion provides a distraction-free environment for participants to focus on completing the activity [70]. Additionally, a purely immersive environment provides a playful aspect that can motivate patients to participate in training [84]. When designing VR interventions, the relationship between presence and immersion in virtual environments should be considered. Immersion, understood as the feeling of “being inside” the virtual environment, measures how much a user feels involved in the environment. On the other hand, presence refers to the perception of “being there” in the virtual environment. Several studies have shown that immersion is closely related to presence, i.e., the more immersive the experience is, the more presence the user experiences [85, 86]. It has been suggested that high levels of presence, related to the level of immersion, explain the effectiveness of training in virtual environments [46]. VR has a high potential to help people overcome mental health problems if high levels of presence are achieved in situations that capture their attention, as described in [87].

Patients with impaired cognitive functions may have a different sense of presence in virtual experiences than healthy people. Low immersion in a VR application was identified as one of the reasons for not obtaining significant improvements in AD patients in one of the studies included in this review [60]. However, the effect of immersion on training outcomes may be weak, as no differences in presence were observed between healthy and MCI groups in the virtual supermarket training conducted by Maeng et al. [66]. This agrees with what Witmer and Singer expressed [88]; a weak but consistent positive relationship exists between presence and task performance in VEs. Considering Howard’s model [89], in VR, presence can increase users’ autonomous motivation by making tasks more meaningful and satisfying, which, in turn, can increase their engagement with the activity. Despite this, more studies are still needed to demonstrate this and present commensurate results.

Visual or auditory help messages have been identified to increase immersion in VR applications and improve iADLs [66, 68]. Auditory cues were provided in a virtual apartment training study [51], while visual cues such as arrows and landmarks were found to assist AD and MCI patients during navigation exercises in a virtual environment [90]. Written and/or verbal instructions have also been shown to be successful in patients with disorganization problems [91], and feedback through verbal reinforcement increases confidence during exercises [64] and motivates the participant [53]. In the study of Park et al. [63], several ADLs were simulated with mixed reality, to which sensory feedback and proprioception were added.

Immersive VR systems include head-mounted displays (HMDs) and hand controls that may cause discomfort to some participants. Non-immersive systems are less likely to cause discomfort in older adults [92], but some studies have reported difficulties using a computer mouse to perform cooking tasks in non-immersive VR interventions [51]. This is related to cybersickness, a potential problem with highly immersive VR interventions, as reported by Zhu et al. [68]. A usability test (SSQ) administered at the beginning of the experiment could help identify participants with increased sensitivity and exclude them from the study [68]. Gradual increases in the duration and difficulty of VR training sessions have been recommended to reduce the incidence of cybersickness [93, 94]. According to the study of Kourtesis et al. [95], the maximum duration for an immersive VR session should range between 55 and 70 min, to avoid experiencing VR-induced symptoms and effects. The session duration of the studies included in our review did not exceed 60 min. No study reported adverse effects except the study of Zhu et al. [68]. In the study, although the duration of each session was 30 min, eight participants had mild cybersickness symptoms in the first 4 activities (measured via the Simulator Sickness Questionnaire [96]), but after the fifth intervention, there were no reports of simulator disease.

Nevertheless, strategies can be applied to mitigate these effects. For example, HMD manufacturers suggest taking short breaks to alleviate the effects of VR, although evidence supporting this recommendation is limited. The study of Szpak et al. [97] does not support the idea that short pauses are effective in mitigating after-effects, as participants reported that these pauses negatively affected their perceived performance in VR compared to longer continuous exposures. More research is needed to determine optimal break durations and improve training programs. Cargenie et al. [98] suggested modifying visual motion cues in VR environments. For example, by tilting visual content on the VR screen, users have to tilt their head and maintain proper viewing while interacting with the app. Haptic feedback, such as vibrations or haptic resistance, could be a consideration to improve interaction and minimize these effects and even redesign HMDs depending on user needs[99].

### Limitations

Based on the studies reviewed, VR interventions have the potential to improve cognitive function in people with mild cognitive impairment and dementia. However, there are several limitations to consider when interpreting the results. One limitation is the variability in the number of sessions, duration, and weekly frequency, making comparing results between studies difficult. In addition, the different instruments used to assess the cognitive domains in the studies also make it difficult to analyze and generalize the results.

Although the interventions have positive effects, they have not been reflected in significant changes in one of the most commonly used scales to assess iADLs, such as the Lawton scale [74]. However, this may be due to the low sensitivity of the test and the ceiling effect reported in other studies [64, 74, 100]. Additionally, long-term follow-up studies to assess the lasting effects of these interventions are lacking. The few studies that have conducted follow-up evaluations, such as the study of Foloppe et al. [53], reported sustained effects up to 6 months after the intervention.

The studies reviewed included individuals with different diagnoses (MCI, MD, AD), which may affect the effectiveness of the interventions. Therefore, caution should be exercised when generalizing results to other populations. Despite these limitations, the evidence suggests that VR interventions may be a promising tool for cognitive training in people with mild cognitive impairments. Future studies with larger sample sizes, standardized cognitive assessments, and longer follow-up periods are needed to further evaluate the effectiveness of these interventions.

Another limitation to be considered in VR interventions for cognitive training is the need for more analysis of the type of motivation that interventions simulating iADLs generate in the participant. Howard’s Self-Determination Theory (SDT) [89] proposes that motivation to engage in a task can be autonomous or controlled. Autonomous motivation occurs when an individual feels that he or she has choices and personal relevance to the task, whereas controlled motivation is based on external pressure or rewards. Studies have shown that interventions that align with autonomous motivation are more effective and have longer-lasting effects than those that rely on controlled motivation. Therefore, it is essential to design VR interventions that provide a sense of autonomy, choice, and personal relevance, such as those that allow users to select and customize tasks or goals.

### Implications for practice

The implications for the practice of VR interventions in cognitive rehabilitation are multifaceted. Motivating participants to engage in tasks is critical for success, as previous studies have shown a high likelihood of dropout [66], possibly due to discomfort or a lack of openness to treatment [101]. It is also important to personalize and tailor interventions to each participant to increase satisfaction, quality of life, and affect [61] while reducing stress, depression, and apathy [61, 68]. The involvement of caregivers is also crucial, as they can promote active participation during and after these types of interventions [53]. As previously mentioned, information on the efficacy of VR interventions is provided in Howard’s Self-Determination Theory (SDT) [89], as studies have shown that interventions that align with autonomous motivation, such as those that provide a sense of autonomy, choice, and personal relevance, are more effective and have longer-lasting effects than those that rely on controlled motivation [53]. Therefore, it is essential to promote and motivate participation, possibly through lectures and demonstration meetings, to reduce the number of dropouts and increase engagement [68].

Another noteworthy aspect is that occupational therapists, psychologists, and clinical neuropsychologists performed most of the interventions in the studies included in this review, ensuring that participants receive appropriate cues and corrections tailored to their clinical condition and based on technical criteria. Although many VR-based applications do not require the intervention of a therapist or family member, studies have shown that satisfaction with cognitive function performance is higher with therapist guidance and accompaniment [59, 102].

Postintervention evidence has demonstrated a likely direct transfer of knowledge to real-life settings, underscoring the potential benefits of VR-based interventions for improving activities of daily living [58]. However, it is important to note that transfer to other daily activities may not always be clear-cut [53]. The Lawton scale, which was used in several reviewed studies, has shown low sensitivity for assessing changes in ADLs. Therefore, the use of other tests, such as the Bayer ADL scale (B-ADL) or the Interview for the Assessment of the Quality of Life in Dementia (QoL-AD), may be more appropriate for this purpose [103]. These scales show greater discrimination than the MMSE in diagnosing dementia in a population with low cultural status and are not influenced by age, education, sex, or country of origin [103]. In addition, the Pfeffer Functional Activities Questionnaire, which assesses each activity on a scale of 0 to 3, may allow greater sensitivity in assessing ADL changes.

### Implications for research

As a common note to many of the reviews found in the literature, we insist on the need for studies of higher methodological quality (RCTs), together with an increase in sample size. There are studies [55, 104] that recommend the use of a passive control group (PCG), since its absence does not allow evaluation of the true therapeutic effect of the variable throughout the period of training or clearly establish its feasibility, acceptability, and tolerability [68]. On the other hand, other studies, such as that of Kang et al. [61], also recommend including an active control group (ACG) to help confirm the clinical efficacy of the intervention. We must also note the need for homogeneous outcome measures in the different studies to perform meta-analyses that give us a better quantitative measure of effect sizes. All this will undoubtedly provide us with more scientific evidence on the role of iADL-VR in cognition.

The neuropsychological tests used to evaluate the participants in the studies have been very heterogeneous, and not all of them have focused on the measurement of EFs, which seem to play a crucial role in the correct performance of iADLs. For this reason, studies should use tests that measure cognitive improvements in EF. However, as Marino noted, it is difficult to perform an adequate neuropsychological evaluation of EFs due to the many functions and capacities involved [105]. That is why we recommend establishing adequate follow-up periods to determine if there is real transfer of knowledge of the skills learned within the experimental set-up. On the other hand, although the studies include a wide variety of measures related to cognition, such as functional memory, memory retention, attention, problem solving, executive functions and cognitive flexibility, spatial cognition, verbal memory, working memory, etc., they have not included measures for the evaluation of personality, socioemotional functioning or adaptive behavior, just as the practice guidelines of the American Academy of Clinical Neuropsychology recommend [106]. According to these guidelines, assessments should also include measures to assess personality, social-emotional functioning, adaptive behavior, etc. Only Kang’s study [61] measured (as secondary outcomes) psychiatric symptoms such as affect, apathy, quality of life (QoL) and depression. Masoumzadeh and Moussavi [57] also included measures of depression, showing a decreasing trend of the participants’ depression scores from baseline to post-intervention. Zhu’s evaluation [68] also found significant improvements in perceived stress and depression in both groups at the end of the intervention. Different studies have previously demonstrated the effectiveness of this type of cognitive rehabilitation for improving mental health [107, 108] and for relieving anxiety and depression [109].

Something that the practice guidelines [106] suggest and that has not been done in the included studies, is the conduct of interviews with family members, especially in MCI or MD participants, to achieve a comprehensive neuropsychological evaluation. These interviews would also be of great importance when verifying the transfer of learned knowledge to real life, since many studies have reported that the effects of the intervention do not translate into greater patient autonomy (based on measures such as the Lawton IADL scale, for example).

Another very interesting line is the development of interventions using VR applications (immersive or not) specifically focused on a certain cognitive disorder and thus verifying their effectiveness [63]. The development of iADL-VR applications is also proposed for the rehabilitation of other sudden cognitive disorders, such as the presence of a stroke or traumatic brain injury [110]. It has been verified in this systematic review that sometimes the applications designed are complicated for the groups with greater cognitive deterioration, for which we suggest that the pathology of the patients be taken into account, and tailor-made applications be carried out, considering both personal and clinical characteristics [64] to maximize the cost–benefit. For example, Maeng et al. [66] recommended that easy-to-operate devices and interfaces be developed to improve the usability level of applications. This ensures that the intervention focuses on cognitive stimulation, not sensorimotor or psychosensometric coordination. Otsuka et al. [111] showed that the adequate adjustment of the difficulty of the tasks and the motivational incentives reduces the frustration of patients with MD, contributing to the benefits of rehabilitation. Based on the -decomposition hypothesis-, all daily tasks are complex, but if they are broken down, they can be executed more easily [112]. Varela-Aldás et al. [113] proposed some improvement ideas, especially for purely immersive systems, such as hand tracking incorporated in the most recent models of HMDs, to improve the usability and concentration of participants.

Regarding the participants’ level of education and performance in the different memory tasks implemented, there is no clear evidence of a discernible relationship between them, in the different studies included in the review, so it seems reasonable to conduct studies fully focused on the effects that education can have on performance on memory tasks. For example, in one of the works included in this review [65], no association was found between performance and education, perhaps because the population was healthy. Other studies that have implemented memory tasks in healthy people [9, 114] did not find differences in performance based on the level of formal education either. Participants with more years of education are expected to have greater processing and conceptualization capacity since the volume of gray and white matter in the brain is greater [115]. In one of the studies included in this review [67], significant differences were found with respect to education, taking into account the results of the Cognitive Reserve Index (CRQ) questionnaire. Those adults with a higher CRI-Education score improved more than those with lower CRI-Education (between pre- and post- intervention). However, Man et al. [59] found evidence that those with less education and MD could benefit more from ADL training. This last idea is related to the study conducted by Mondini et al. [116], where the authors concluded that those with low cognitive reserve can benefit more from rehabilitation. Other factors that may influence the neuropsychological evaluation process should also be taken into account. They include socioeconomic, cultural, linguistic or disability issues, among others [106]. For example, Tarnanas et al. [54] indicated in their study that interindividual genetic variability modulates the transfer of training to untrained tasks. Therefore, individual differences in cognitive training outcomes should be addressed in future works.

Although many studies affirmed that the use of VR contextualized in IADLs contributes to improving the motivation of the participants and to retaining patients during the interventions, in none of them has an evaluation of motivation and effort been carried out. Failing that, the introduction of common sense methods to optimize the participant’s performance is interesting. In this sense, only one study [70] included activities (for both groups), such as reading/discussing newspapers and magazines, watching TV documentaries, and creative and painting workshops. The inclusion of such activities can minimize anxiety, physical discomfort, and other factors that may interfere with optimal motivation and effort [106].

It would also be convenient to incorporate the use of fMRI in these investigations to explore the neuronal plastic changes that can positively affect a person’s cognition. The positive findings found by Optale et al. [70] support the theories of plasticity in the neuronal system in patients with impaired cognitive functions. Similarly, there is evidence of these processes in cognitively healthy elderly individuals [117] and those with AD [118]. Furthermore, multidomain training may be more effective in improving neuroplasticity mechanisms [119].

Finally, an aspect that can be analyzed and has not been considered so far deals with people’s social activity as an extension of an iADL (social participation) since these activities are also performed daily [9]. The study conducted by Tomioka et al. [120] found a strong association between participation in social groups and the execution of iADLs independently by the participants. Perhaps the design of VR tasks based on this type of activity would give us a new dimension on the path towards improving independence in performing these tasks [78].

## Conclusions

In recent years, VR has gained popularity as a complementary intervention for treating cognitive impairment. Specifically, VR applications based on instrumental activities of daily living (iADL-VR) have emerged as a promising approach to train, rehabilitate, or stimulate cognitive functions in people with cognitive impairment, Alzheimer’s disease, or healthy adults. This systematic review aimed to synthesize the effects of iADL-VR interventions on cognitive function in older adults and people with cognitive impairment. The results of this review indicate that iADL-VR interventions have the potential to improve cognitive function, with almost all studies showing improvements in some or all of the outcomes after the intervention, generally being greater in the iADL-VR group than in the control group.

Furthermore, the ecological component of these tasks makes them suitable for transferring what has been learned to the real world, emphasizing the potential benefits of these interventions for improving activities of daily living. However, further research with larger and more homogeneous samples and longer follow-up periods is needed to confirm the transferability of these interventions to the real world. The limitations of the current studies must be addressed in future research, including variability in the number of sessions, duration, and frequency of intervention, as well as the use of different instruments for assessing cognitive domains. Future studies should also consider the potential role of factors such as motivation and autonomy support in enhancing the effectiveness of these interventions.

This systematic review highlights the potential of iADL-VR interventions as a promising approach to cognitive rehabilitation, training, and stimulation in older adults and people with cognitive impairment. However, further research is needed to better understand the mechanisms underlying the efficacy of these interventions and optimize the design and implementation of iADL-VR interventions to maximize their effectiveness.

### Supplementary Information

**Additional file 1. Supplement Table A1.** Databases and search terms used

## Data Availability

The datasets generated during and/or analyzed during the current study are available from the corresponding author upon reasonable request.
