# Supplementary material for: Virtual reality applications based on instrumental activities of daily living (iADLs) for cognitive intervention in older adults: a systematic review
Source: J Neuroeng Rehabil. 2023 Dec 19;20:168. doi: 10.1186/s12984-023-01292-8 (PMC10729470; doi:10.1186/s12984-023-01292-8)
Supplement: Supplementary file 1 — Additional file 1. Supplement Table A1. Databases and search terms used [file 12984_2023_1292_MOESM1_ESM.docx]

**Annex I. Supplement Table A1.** Databases and search terms used

| **N°** | **MESH/search terms** | **Database** | | | | | **Total** |
| --- | --- | --- | --- | --- | --- | --- | --- |
|  |  | **Pubmed** | **Scopus** | **IEEE Xplore** | **Apa PsycNet** | **Web of Science** |  |
| 1 | ADL and Virtual | 70 | 197 | 69 | 25 | 189 | 550 |
| 2 | IADL and Virtual | 28 | 46 | 5 | 12 | 37 | 128 |
| 3 | virtual AND (daily OR adl) AND (cognitive OR memory OR executive) NOT (“brain injury") NOT (stroke) AND (rehabilitation OR training OR stimulation OR remediation) | 288 | 955 | 45 | 153 | 246 | 1687 |
| 4 | virtual AND (daily OR adl) AND (dementia OR mild) AND (cognitive OR parkinson OR alzheimer OR memory) NOT ("brain injury") NOT (stroke) AND (rehabilitation OR training OR stimulation OR remediation) | 61 | 173 | 10 | 31 | 67 | 342 |
| 5 | virtual AND (rehabilitation OR training OR stimulation OR remediation) AND (cooking OR kitchen) NOT (brain injury OR stroke) | 56 | 70 | 1 | 22 | 75 | 224 |
| 6 | virtual AND (training OR rehabilitation OR stimulation OR remediation) AND (store OR shopping OR supermarket) NOT "brain injury" NOT stroke | 317 | 366 | 293 | 127 | 693 | 1796 |
| 7 | computer AND cognitive AND (alzheimer OR dementia OR mild) AND (rehabilitation OR training OR stimulation OR remediation) AND (ADL OR daily) NOT ("brain injury" OR stroke) | 289 | 171 | 29 | 18 | 160 | 667 |
| 8 | virtual AND (rehabilitation OR training OR stimulation OR remediation) AND (store OR shopping OR supermarket) AND (dementia OR mild) AND (cognitive OR parkinson OR alzheimer OR memory) NOT ("brain injury" OR stroke) | 23 | 34 | 5 | 6 | 29 | 97 |
| 9 | virtual AND (cook OR cooking OR kitchen) AND (dementia OR mild) AND (cognitive OR parkinson OR alzheimer OR memory) NOT ("brain injury") NOT (stroke) AND (rehabilitation OR training OR stimulation OR remediation) | 6 | 9 | 0 | 3 | 10 | 28 |
| 10 | virtual AND (car OR driving) AND (dementia OR mild) AND (cognitive OR parkinson OR alzheimer OR memory) NOT ("brain injury") NOT (stroke) AND (rehabilitation OR training OR stimulation OR remediation) | 7 | 16 | 0 | 3 | 23 | 49 |
| **Total** | | 1145 | 2037 | 457 | 400 | 1529 | 5568 |
